# Nanoemulgel for Improved Topical Delivery of Retinyl Palmitate: Formulation Design and Stability Evaluation

**DOI:** 10.3390/nano10050848

**Published:** 2020-04-28

**Authors:** Mohammed S. Algahtani, Mohammad Zaki Ahmad, Javed Ahmad

**Affiliations:** Department of Pharmaceutics, College of Pharmacy, Najran University, Najran 11001, Saudi Arabia

**Keywords:** Vitamin A, retinoid, chemical/photo-instability, nanoemulsion, topical delivery, skin permeability, nanoemulgel

## Abstract

Retinyl palmitate is a vitamin A ester belonging to the family of endogenous natural retinoid and used to treat various skin disorders like acne, skin aging, wrinkles, and dark spots, as well as to protect against psoriasis. Despite the known therapeutic benefits of retinyl palmitate, the conventional topical delivery of retinyl palmitate commonly associated with adverse reactions such as skin irritation, redness, excessive peeling, and dryness. Therefore, the current study aims to encapsulate the retinyl palmitate in nanoemulsion then incorporate it into a hydrogel system to improve the topical delivery and stability. Low-energy emulsification method was used for the nano-encapsulation of retinyl palmitate. The phase behavior study was used for the investigation and the optimization of the formulation. The droplet size of the optimized nanoemulsion was in nano dimension (16.71 nm) with low polydispersity index (PdI) (0.015), negative zeta potential (−20.6 mV). It demonstrated the influence of vortexing on droplet size and PdI during nanoemulsion preparation. The retinyl palmitate loaded nanoemulgel delivery system exhibited significant improvement (*p* < 0.05) in skin permeability after topical application. Employment of the nano-encapsulation approach afterward dispersion into hydrogel system for the development of a topical delivery system of retinyl palmitate resulted in improvement in its UV and storage stability as well.

## 1. Introduction

Retinyl palmitate (RT) is a lipophilic compound of the retinoid class used to treat various skin disorders such as acne, skin aging, wrinkles, and dark spots, as well as to protect against psoriasis and ichthyosis [1]. Additionally known as vitamin A ester, RT belongs to the family of endogenous natural retinoids that help maintain healthy skin, hair, and mucous membranes. RT works through exfoliating the surface layer of the skin, thus speeding up cell turnover and making the skin look fresher, smoother, and younger by increasing the skin’s elasticity and decreasing the peroxidation of lipids in the skin. Additionally, RT increases skin moisture and decreases skin wrinkles [1,2]. Applied topically, RT acts as an antioxidant on the skin, preventing tissue atrophy secondary to age-associated loss of collagen. Furthermore, RT has demonstrated antimicrobial activity against bacteria that cause acne and anti-inflammatory effect as well [3].

Despite the potential therapeutic benefits of RT, there are some properties that hinder its efficacy and cause side effects [2]. Conventional formulations containing RT have demonstrated chemical/photo-instability, poor aqueous solubility, increasing toxicity at higher concentrations, and the potential to cause skin irritation, redness, excessive peeling, and dryness following their administration [3,4,5]. Therefore, novel formulations of RT are needed that improve the topical efficacy of RT in treating various skin disorders.

The encapsulation of various lipophilic nutraceuticals/pharmaceuticals/cosmeceuticals inside lipid-based nanocarrier systems has been demonstrated to protect from photo/chemical degradation, improve the aqueous solubility, and allow deeper skin penetration of similar active ingredients [6,7]. Solid lipid nanoparticles [8], nanostructured lipid carriers [9], nanocapsules [10], and nanoemulsions (NEs) [11] are examples of lipid-based systems that have been proven to decrease drug degradation, improve drug targeting, and enhance the efficacy of retinoids in the treatment of skin disorders [12]. Among the various lipid-based formulations designed to deliver retinoid compounds, NE-based drug delivery systems have been identified as the most feasible and economical method of topical therapy for various skin disorders. Through research advancements using homolipids and heterolipids as excipients, NE formulations have gained much attention for their ability to enhance the topical efficacy of otherwise poorly permeable retinoid compounds. NEs have demonstrated wide compatibility with different retinoid compounds, surfactants, and oil systems, and they are also easy to process and manufacture, thus generating further interest in NEs as drug carriers in the development of various topical formulations.

NEs consist of colloidal oil droplets, ranging in size between 20 and 200 nm, dispersed in an immiscible aqueous medium [3]. The ability to load drugs using solvent-free low-energy preparation methods and the complete entrapment/encapsulation efficiency (100%) of these therapeutics are both significant advantages of NE-based drug delivery systems compared to other lipid-based methods. However, the low viscosity of NEs makes their direct topical application inconvenient and modulates the skin permeation profile. The incorporation of NEs into hydrogel systems commonly referred to as nano-emulgel (NEGs), has improved the topical efficacy of various, otherwise poorly permeable, therapeutics. Encapsulation of RT within a NE system both protects the RT from degradation and allows for deeper skin permeation. Moreover, the incorporation of the RT-loaded NEs into hydrogel systems (as a secondary vehicle) results in enhanced control of RT release from the delivery vehicle, ultimately minimizing the chance of skin irritation and improving patient compliance.

The current study aimed to improve the delivery of topically applied RT through employing a NE-based delivery system. A NE loaded with RT was incorporated into a hydrogel system as a novel NEG to enhance the permeation of RT through the skin and to protect it from degrading, thereby improving the biopharmaceutical performance of the drug and minimizing the various side effects of the conventional cream and gel products currently available.

## 2. Materials and Methods 

### 2.1. Materials

The RT used in this study was purchased from Cayman Chemicals (Ann Arbor, MI, USA). The Kolliphor^®^ EL, Kolliphor^®^ HS 15, Tween 20, triethanolamine, and glycerol were purchased from Sigma Aldrich (Taufkirchen, Germany). The Caproyl 90 (propylene glycol monocaprylate) and Transcutol HP were purchased from Gattefosse (Saint Priest, France). Captex^®^ 355, Capmul^®^ MCM, and Capmul^®^ PG-12 were provided by the Abitec Corporation (Columbus, OH USA). Water was obtained using a Milli-Q water purification system (Millipore; Billerica, MA, USA). All other chemicals used in this study were analytical grade reagents.

### 2.2. Screening and Optimization of the Formulation Components

The screening and optimization of formulation components used for the development of the drug loaded NEs depended on the phase solubility of the payload and the phase behavior between the formulation excipients. 

The phase solubility of RT in both the lipid phase (similar to oils) and Smix phase (mixture of surfactants and co-surfactants) was investigated using the shake-flask method. The lipid phase was screened based on the maximum solubilization capacity of RT. The various surfactants were chosen based on their emulsification potentials for the lipid phase and were screened by their maximum solubilities of RT [13]. The co-surfactants used were chosen based on their respective maximum nanoemulsifying regions, which were obtained by constructing phase diagrams for the selected surfactant and oil systems [14]. 

The phase behaviors of the different components comprising the nanoemulsion system were determined through studying their phase diagrams, which were constructed on ternary plots using the aqueous titration method [15]. The surfactant was solubilized in an oil system at various ratios and continuously titrated through the addition of drop-by-drop distilled water. Following the addition of each water drop, the sample was vortexed extensively and then observed for any milky appearance or other phase behavior. The percentage proportion of the oil, Smix, and water phase were determined, and phase diagrams constructed to examine the phase behavior between the NE component. 

### 2.3. Preparation and Characterization of the Nanoemulsions

The RT-loaded NEs were prepared through the low-energy emulsification technique by uniformly mixing optimized oil and Smix phases with a vortex mixer, using the optimum ratio of components obtained from the phase diagrams [14,15]. After uniform miscibility of the oil and Smix phases, purified water was immediately added as an aqueous phase and then vortexed to achieve a transparent colloidal dispersion in the form of a NE. These thermodynamically stable formulations of NE were then characterized for droplet size, polydispersity index (PdI), zeta potential, and percentage of contained RT.

#### 2.3.1. Thermodynamic Stability Study

The RT-loaded NEs were subjected to various stress tests, including heating-cooling cycles, freeze-thaw cycles, and centrifugation tests [16]. The NEs were monitored for any physical instabilities during stress testing (indicated by phase separations, drug precipitations, or color changes), to exclude those NEs from further investigation and characterization.

#### 2.3.2. Viscosity

The viscosities of the optimized, RT-loaded NE systems were determined without further dilution, using a Bohlin rotational viscometer (BohlinVisco 88; Malvern Instruments Ltd., Malvern, UK) at 25 ± 0.5 °C [17].

#### 2.3.3. Drug Content Analysis

To determine the RT content of the various optimized NE systems, each sample (100 µL) was diluted with methanol at a ratio of 1:1000 [18] and then analyzed using a UV spectrophotometer with a λmax of 325 nm.

#### 2.3.4. Droplet Size Analysis

The droplet size distributions and PdIs of the optimized NE systems were investigated in triplicate by dynamic light scattering using a Zetasizer (ZS90; Malvern Instruments Ltd., Malvern, UK). Each sample (100 µL) was diluted with distilled water at a ratio of 1:100 before the analysis [19].

#### 2.3.5. Zeta Potential Determination

The zeta potentials (ζ) of the optimized NE systems were assessed through laser Doppler anemometry using a Zetasizer (ZS90; Malvern Instruments Ltd., Malvern, UK). Each sample (100 µL) was diluted with distilled water at a ratio of 1:100 before the analysis [19].

### 2.4. In-Vitro Drug Diffusion Study

The in-vitro diffusion rates of RT in the optimized NE systems were evaluated using the dialysis bag method [20]. Dialysis bags were filled with 1 mL of the various RT-loaded NE systems and suspended in release medium (PBS, pH 7.4) at 37 °C. At fixed time intervals, 1 mL aliquots were extracted from the bags and immediately replaced by an equal amount of release medium. Aliquots were analyzed for the content of RT by UV-spectroscopy at λmax 325 nm. Experiments were performed in triplicate to evaluate the diffusion profile of RT from optimized NE formulations.

### 2.5. Preparation and Characterization of Nano-Emulgel

NEGs containing the optimized NE systems were prepared using Carbopol 940 (0.5% *w*/*w*) for topical administration [21]. Accurately weighed amounts of Carbopol 940 were dispersed in distilled water and keep overnight to achieve uniform swelling. Glycerin as humectant was incorporated into the dispersion system in order to provide a smooth and soothing effect. Triethanolamine was added into the dispersion system drop-by-drop to neutralize the pH to 5.5, resulting in instant conversion to a hydrogel system. Finally, the optimized NE systems were homogeneously incorporated into the placebo gel to obtain RT-loaded NEGs (RT-NEGs). 

The pH, rheology, spreadability, extrudability, and drug content uniformity of the various RT-NEGs were evaluated as follows:

#### 2.5.1. pH Analysis

Accurately weighed (2.5 g) quantities of RT-NEGs were diluted with known volume of distilled water (25 mL). The pH of the RT-NEGs was determined using a digital pH meter (PP201; Ezodo, Taipei City, Taiwan) after suitable dilution (10% *w*/*v*). The pH of each NEG system was measured in triplicate and considered as the average of the three values [17].

#### 2.5.2. Rheology

The rheological properties of the placebo gel and RT-NEGs were investigated by rotational viscometer at 25 ± 0.5 °C (Bohlin Visco 88; Malvern Instruments Ltd., Malvern, UK). The RT-NEGs were tested to determine their respective shear-stress profiles (15–200 to 200–15 Pa in 60 steps with an equilibration time of 10 s at each step) and thixotropic behaviors during simulated topical administration [17]. Bohlin R6.51.03 software was used to calculate the various rheological profiles. 

#### 2.5.3. Spreadability

The spreadability of the various RT-NEG formulations was determined by compressing an accurately weighed (1.0 g) quantity of sample under a glass plate of known weight. The spreading area of each sample was measured, and the results were expressed as a function of the spreading area to applied mass [22].

#### 2.5.4. Extrudability

To determine the extrudability characteristics of NEG formulation, a sealed collapsible tube containing the formulation was enforced firmly at the folded end. Then, as the cap opened, the gel preparation was extruded because of the force applied. The force applied to extrude a fixed amount of gel in specific time-interval was assessed to optimize the extrudability behavior of NEGs system [18]. 

#### 2.5.5. Drug Content Uniformity

A total of 500 mg of the formulations were sampled from random portions of each NEG formulation. Samples were extracted through the addition of methanol as an extracting solvent for 30 min with intermittent vortexing. Extracts were centrifuged at 3000 rpm for 15 min, after which the supernatants were filtered using a syringe filter with a membrane pore size of 0.45 µm. After dilution with methanol, the amount of RT in each extract was determined using a UV spectrophotometer (λmax 325 nm). Each analysis was performed in triplicate, and the content uniformity of RT was assessed as the average content in terms of percentage [23].

### 2.6. In-Vitro Skin Permeation and Deposition Study

The in-vitro skin permeation of RT from the NEG systems was evaluated using a static Franz diffusion cell [24]. A Franz diffusion cell is divided into two compartments (donor and receptor compartments) and a sample of shaved, excised dorsal skin from Wistar rats was mounted between these two compartments. The RT-NEG sample was applied to the donor compartment, while the receptor compartment was filled with release medium (PBS, pH 5.5) and the whole assembly was maintained at 37 °C. Aliquots were collected at different time intervals (0, 0.25, 0.5, 1, 2, 3, 4, 6, 8, 10, 12, and 24 h) and replaced by an equal volume of receptor media. The aliquots were analyzed using a UV spectrophotometer (λmax 325 nm) to elucidate the cumulative amount of drug that had permeated the skin by the various time intervals. 

In-vitro drug deposition within the skin was evaluated using the same skin samples utilizing the tape-stripping technique [25]. The skin samples were unclipped from the Franz diffusion cells after 24 h of the permeation study and then washed with PBS. Cellophane tape was used for the tape stripping of skin. The first strip of tape was discarded due to the fact they potentially contained drug that was adhered to the surface of the skin sample. Approximately 10 strips were used in the removal of the entire subcutaneous (SC) layer of skin, in a manner that utilized the maximum area of tape. The treated skin samples and tape used for the stripping procedure were both then chopped and incubated in ethanol to completely extract the RT. Afterward, samples were sonicated for 15 min and then centrifuged at 3000 rpm for 15 min. The extracted samples were analyzed by UV spectroscopy at λmax 325 nm to measure the amount of RT deposited in the skin. This procedure for quantifying the skin permeation was then repeated for the RT-gel (accurately weighed amounts of RT dissolved in the small quantity of propylene glycol and dispersed into placebo gel to obtain RT-gel of strength 1% *w*/*w*) and RT creams (accurately weighed amounts of RT in the required quantity of cream base [composed of PEG 4000, PEG 400, lanolin, glyceryl monostearate, and poloxamer 188] to obtain a RT cream at a concentration of 1% *w*/*w*) in order to compare the skin permeation of RT with that of the developed NEG systems. 

### 2.7. Stability Study

#### 2.7.1. Physical Stability

To evaluate the storage stability of RT-loaded NEs prepared using different vortex conditions (0, 1, 3, and 5 mins), samples were added to Eppendorf tubes and stored for three months at 25 °C. The effects of sample storage on the physical appearance, mean droplet size, and PdI were evaluated at regular time intervals (0, 15, 30, 60, and 90 days).

In addition, RT-loaded NEs incorporated in hydrogel system as RT-NEGs were also evaluated for storage stability at 25 °C for a period of 3 months. The samples were placed in collapsible tubes and withdrawn at each time interval (0, 30, 60, and 90 days) to observe the changes in physical appearance, pH, rheological properties and drug content [26].

#### 2.7.2. UV Stability

The UV stability of RT, both in its pure state and the NEGs, was determined by keeping the sample in a UVA irradiation chamber (λ ~ 320–400 nm). Samples containing 60 mg of RT were placed in petri dishes at a distance of approximately 15 cm from the UV lamp (10 W; Philips, Amsterdam, The Netherlands) and irradiated with UV light for a period of up to 24 h [27]. The concentration of RT in each sample was calculated at different time points (0, 2, 4, 6, and 24 h) using UV–visible spectrophotometric analysis with a λmax of 325 nm.

### 2.8. Statistical Analysis

The statistical analysis was carried out through software GraphPad Prism 6.0 (version 6.05; GraphPad Software Inc., San Diego, CA, USA). Data were analyzed utilizing one-way ANOVA followed by Tukey’s multiple comparisons test. The *p* < 0.05 was considered as statistically significant.

## 3. Results and Discussion

### 3.1. Preformulation Study

RT, a yellowish viscous substance at room temperature, was found to be highly miscible with Captex 355 (> 1000 mg/g), Capryol 90 (305.12 ± 4.61 mg/g), and Transcutol HP (501.45 ± 4.57 mg/g). RT also exhibited good miscibility with Kolliphor EL (> 432.01 ± 3.80 mg/g) and Tween 20 (> 128.76 ± 3.64 mg/g) (Table 1). The relatively high solubility of RT in Captex 355 in the oil phase is potentially due to the high lipophilicity and low Hydrophile-Lipophile Balance (HLB) value (< 1) of Captex 355 compared to Capryol 90 (HLB = 5). 

**Figure 1:**
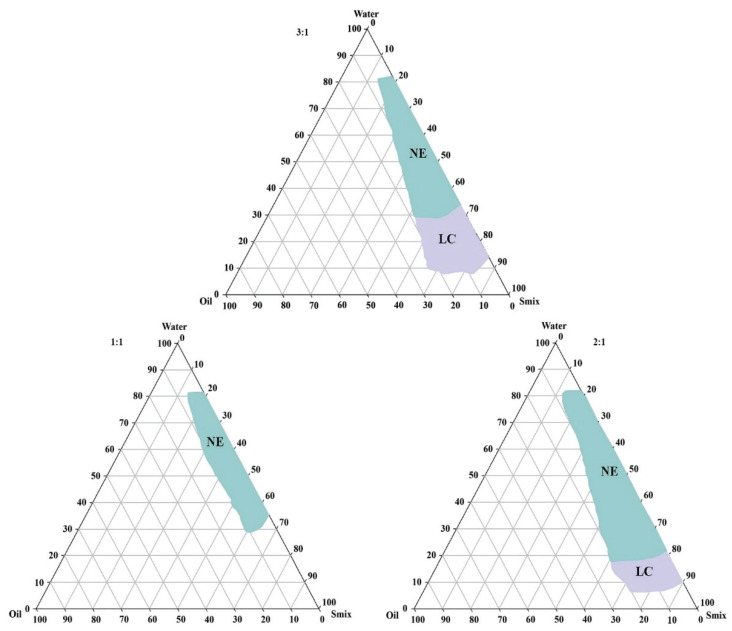
Pseudo-ternary phase diagrams demonstrating the influence of the Smix ratio (1:1, 2:1, and 3:1) on the nanoemulsion (NE) and liquid crystal (LC) regions.

The phase behavior study was performed to investigate the influences of various formulation components on the formation of the NEs. An oil phase with maximum drug solubility is desirable for the development of a NE as it permits the formulation to be loaded with a high drug concentration. The specific gravity and HLB value of the oil phase is also important with regards to the development of a NE via the low-energy emulsification method. The oil system was optimized by combining medium-chain monoglycerides (Capryol 90, HLB 5) and medium-chain triglycerides (Captex 355, HLB < 1) in a 2:1 ratio to achieve an oil phase with optimum HLB and desirable loading of RT in the NE through the low-energy method. The HLB value of the surfactant system serves as a critical guide in terms of monitoring the mixing of the aqueous and oil phases during the transformation into an NE. Non-ionic surfactants with higher HLBs and lower critical micelle concentrations (CMCs) have more stable micelles, and are thus more suitable for drug delivery systems [28]. Co-surfactants are used in combination with surfactants to bestow flexibility on the surfactant film around the NE. Furthermore, co-surfactants play a crucial role in overcoming the repulsive forces and fluidity of the aqueous and oil phases, respectively [28]. The relationship between the phase behavior of an NE and the surfactant-co-surfactant mass ratio (*Km*) can be explained with the help of a pseudo-ternary phase diagram [16]. The system underwent transitions from transparent to translucent to opaque via rearrangements in the ingredients within the NE phase, which affected the light-scattering behavior of the system. The NE area was used to evaluate the *Km*, such that the larger the area of the NE region the greater the nanoemulsification efficiency of the system [29]. Therefore, phase diagram studies were performed using the oil phase (Capryol 90 and Captex 355 in a 2:1 ratio), Smix phase (Kolliphor EL and Transcutol HP), and water phase through aqueous titration. Pseudo-ternary phase diagrams were constructed and the influence of *Km* on the area of the NE region and phase behavior are depicted in Figure 1.

In this study, the suitability of commonly used, generally recognized as safe (GRAS) grade non-ionic surfactants (Tween 20, Kolliphor^®^ EL, and Transcutol HP) were investigated for potential use in the development of a RT-loaded NE. Transcutol HP is a widely used co-surfactant in the development of NEs for topical application. Therefore, Transcutol HP was investigated by preparing Smix combinations with Tween 20 and Kolliphor EL. The phase behaviors of NE preformulations containing these various components are summarized in Table 2.

Based on the results obtained from the preformulation study, the oil phase (Capryol 90 and Captex 355 in a 2:1 ratio), Smix phase (Kolliphor EL and Transcutol HP), and water phase concentrations were optimized and a RT-loaded NE was prepared for further investigation.

### 3.2. Preparation of the Nanoemulsion

RT (30 mg) was dissolved in the homogenous oil (Capryol 90 and Captex 355, 2:1 ratio) and Smix phases (Kolliphor EL and Transcutol HP, 2:1 ratio) by vortex mixing. The water phase was then immediately added and the solution was vortexed to obtain a transparent dispersion system NE with a drug loading of 30 mg/mL.

#### 3.2.1. Effect of Vortexing Time on the Nanoemulsification

**Figure 2:**
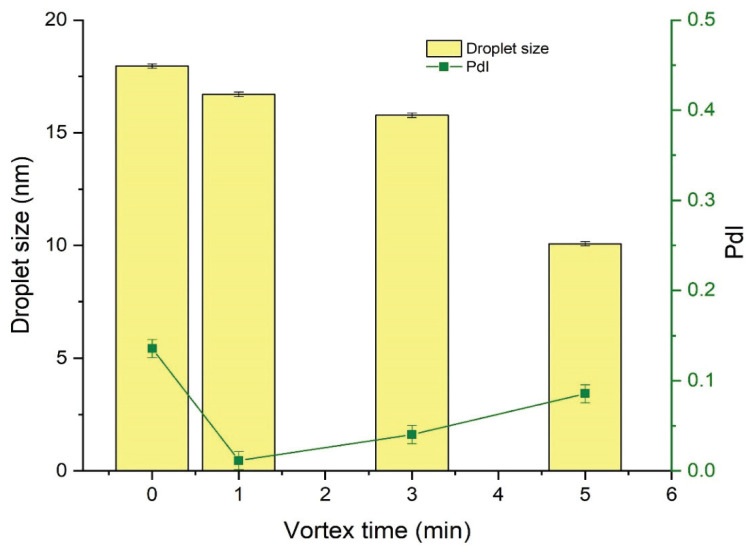
Effect of vortex time on the droplet size and polydispersity index (PdI) of nanoemulsion preparations. The increase in vortexing duration provided more energy to further minimize the droplet size of developing nanoemulsion system. Different compositions of RT-loaded NEs, with oil concentrations ranging from 10–20%, Smix concentrations (2:1) ranging from 45–50%, and water concentrations ranging from 30–45% were prepared and characterized in terms of thermodynamic stability, percentage transmittance (%T), viscosity, drug content, droplet size, PdI, and zeta potential (Table 3).

The effect of the vortexing time on the droplet size and PdI of NEs was investigated by preparing a series of NEs of fixed composition that were then vortexed for 0 (no vortexing), 1, 3, or 5 min. Results indicated that vortexing time was inversely related to the droplet size of the subsequent NE (Figure 2). Vortexing time also influenced the PdI of the NE, and decrease the value of PdI to a certain extent only afterward start increasing as shown in Figure 2.

### 3.3. Characterization of Retinol-Loaded Nanoemulsions

The NE formulations used in the phase diagram study were subjected to thermodynamic stability testing, which included heating-cooling cycles, centrifugation tests, and freeze-thaw cycles. All of the tested formulations (NE1-NE4) demonstrated no evidence of emulsion instability (such as creaming, cracking, or coalescence) and successfully passed the stress tests. The %T of all formulations (NE1-NE4) was determined to be > 95%, indicating the tested formulation systems were in a state of fine dispersion (Table 3). Additionally, the tested formulations were completely transparent in appearance, inferring droplet sizes were in the submicron range due to the minimal light scattering.

Viscosity measurements on the selected formulations (NE1-NE4) were carried out at ambient temperature (25 °C), results are displayed in Table 3. NE2 demonstrated the highest viscosity (89.22 ± 1.95 cp), while NE4 exhibited the lowest (77.48 ± 1.73 cp). The results demonstrated that the viscosity of the RT-loaded NEs (NE1-NE4) remained constant with increases in shear rate, and the NEs exhibited Newtonian fluid behavior [30].

The percentage of RT content in each optimized NE (NE1-NE4) was quantified using UV-spectrophotometric analysis and was found to be approximately 99.0% in all cases, as shown in Table 3. 

The droplet size distribution, surface charge (zeta potential), and PdI represent the main features that need to be considered when formulating a stable NE system. These key parameters of NEs affect their bulk properties, product performance, stability, and appearance [31]. The mean droplet size, PdI, and zeta potential of the varying NE formulations (NE1-NE4) are shown in Table 3 and illustrated in supplementary Figures S1, S2, S3, and S4. The oil concentration significantly affected the mean droplet size of the NE. As observed in Table 3, increasing the oil concentration from 10% to 20% while keeping the Smix phase concentration (45%) and RT loading (30 mg/mL) constant, increased the mean droplet size by a factor of 4.5 (NE1 compared to NE4). Similarly, mean droplet size increased by a factor of 2.5 when the oil concentration was increased from 15% to 20% while keeping the Smix concentration (50%) and RT loading (30 mg/mL) constant (NE2 compared to NE3). The PdI of the selected formulations (NE1-NE4) varied between 0.015 and 0.61, following the order NE1 > NE2 > NE3 > NE4. This confirms that percentage of oil concentration in formulation composition greatly influences the size distribution of NE. Zeta potential is defined as the potential difference between the surface of a tightly bound layer and an electroneutral region of the system [30] and measures the charge on the surface of dispersed globules in a NE system. The concentration of oil and Smix did not significantly affect the value of the zeta potential (Table 3), potentially due to the constant amount of RT loading (30 mg/mL) present in each composition system. The variation in the value of the zeta potential as the RT loading changes is a result of the RT dispersing over the surface of the oil droplets, rather than within them [32]. For topical applications, the ideal globular size of NEs is less than 50 nm, with a PdI value < 1, thus providing a larger surface area and leading to deeper penetration by a higher amount of the payload [33]. Considering this, the NE2, NE3, and NE4 formulations were selected for use in the in-vitro drug release study.

### 3.4. In-Vitro Drug Release Study

**Figure 3:**
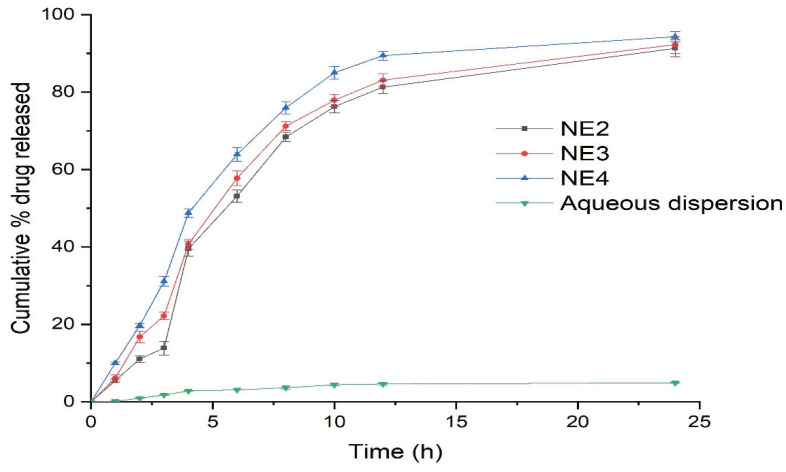
In-vitro drug release study comparing retinyl palmitate (RT)-loaded nanoemulsions to the aqueous dispersion of pure RT using the dialysis bag technique.

The in-vitro release of RT from the selected NE formulations (NE2-NE4) was performed using the dialysis bag technique for the duration of 24 h (Figure 3). The release of RT from the NE formulations was compared to the aqueous dispersion of pure RT.

**Figure 4:**
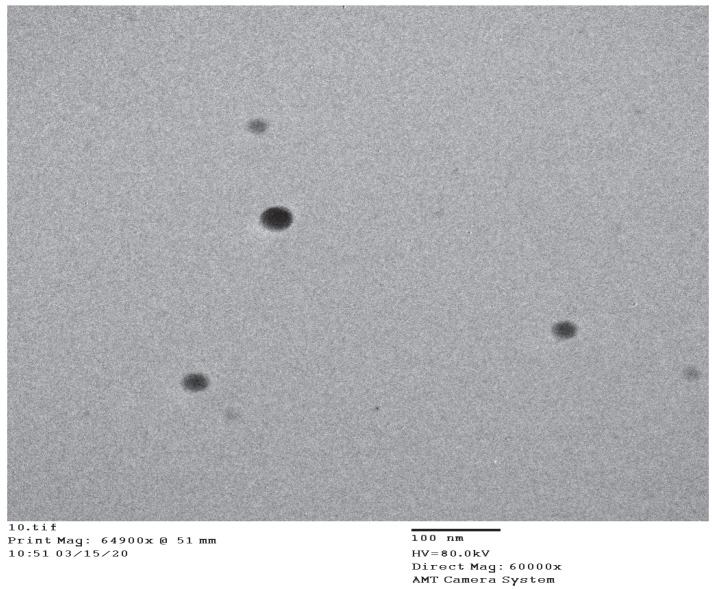
Droplet morphology of optimized nanoemulsion (NE4) system under transmission electron microscopy.

The release of RT from the NE formulations was significantly higher than the aqueous dispersion of pure RT. Specifically, the RT release from the NE systems (NE2-NE4) was in the range of 89–94%, compared to 9.89% from the aqueous dispersion within 24 h. The NE4 formulation system demonstrated the maximal release of RT, presumably due to the smaller mean droplet size of this system compared to the other NEs (NE2 and NE3). Using these results, NE4 was selected as the optimal NE for conversion into a NEG system and droplet morphology of NE4 characterized through transmission electron microscopy (TEM) is shown in Figure 4.

### 3.5. Preparation and Characterization of the Nano-Emulgel

The NE4 system was introduced into an aqueous dispersion of Carbopol 940 (0.5% *w*/*w*) and glycerol (2% *w*/*w*), converting it into an RT-containing NEG system with a strength of 1% *w*/*w*. The dispersion was then neutralized to a pH of 5.5 by adding 2–3 drops of triethanolamine.

The pH of topical formulations is ideally in the range of the skin’s pH, so as not to cause disturbances to the skin acid mantle. The pH of the developed RT-NEG was 5.53 ± 0.06, which is similar to that of the skin acid mantle.

**Figure 5:**
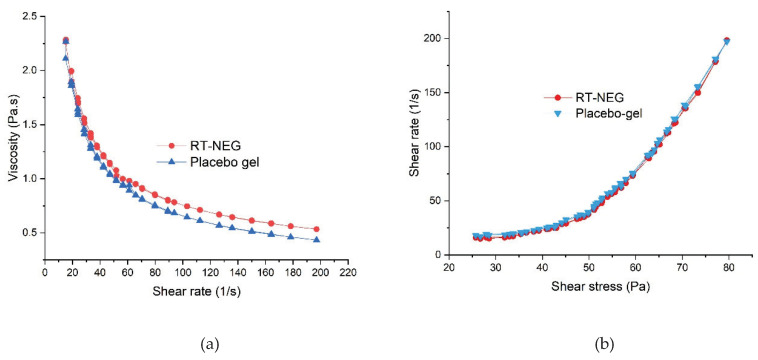
Rheological behavior of the retinyl palmitate-loaded nano-emulgel (RT-NEG) and placebo gel, demonstrating (**a**) viscosity versus shear rate and (**b**) shear rate versus shear stress.

The rheological profiles of the placebo gel and NEG were determined using a parallel plate rotational viscometer. The placebo gel and developed NEG both exhibited similar rheological behavior (Figure 5). As illustrated in Figure 5, the developed NEG system underwent gel-to-sol transition and exhibited shear-thinning following the application of shear stresses. However, slow recovery to gel began upon the removal of this stress, indicating the developed NEG system exhibited non-Newtonian, pseudo-plastic behavior (shear-thinning) with thixotropic properties [30]. Pseudo-plastic behavior of gel formulations is convenient for application and desirable for the topical delivery of therapeutics [34].

Good spreadability helps to achieve uniform application of topical gels [35] and thus the spreadability factor (cm^2^/g) is one of the most important qualities to consider when developing a semisolid pharmaceutical formulation intended for skin application [36].

Results demonstrated that increases in the weight as applied force increased the spreading area of the developed RT-NEG, RT-gel and placebo-gel. There was no significant difference in the spreadability factor of the RT-NEG or the RT-gel when compared to the placebo gel (Table 4). Additionally, the RT-NEG demonstrated good extrudability from the container for convenient use by the consumer. Drug content analysis was performed on the developed RT-NEG and demonstrated uniform dispersion of RT within the NEG system. Specifically, the percent uniformity of RT in the NEG system was calculated to be 99.32 ± 0.121% while the RT content was 98.72 ± 1.15%.

### 3.6. Skin Permeation and Deposition Study

A comparative ex-vivo drug deposition study was performed on the RT-NEG, RT-gel, and RT cream using Franz diffusion cells equipped with the excised skin of albino rats. Table 4 displays the results of this skin permeation and deposition study. The amount of RT deposited in the deeper layer of skin by RT-NEG (835.56 ± 19.59 µg/cm^2^) was more than 4-fold higher than that deposited by the RT-gel (204.29 ± 10.1 µg/cm^2^) or RT-cream (173.35 ± 6.29 µg/cm^2^). The percutaneous drug flux (Jss) of RT from the RT-NEG system (16.85 ± 0.4) was more than double the Jss of RT from the RT-gel (8.54 ± 0.42) and RT-cream (7.33 ± 0.47). Additionally, the permeability coefficient (K_p_) of RT from the RT-NEG system (3.37 ± 0.08) was also found to be more than double that of the RT-gel and RT-cream. The permeation enhancement ratio (ER) of RT released from the RT-NEG system compared to the RT-gel and RT cream were 1.97 ± 0.07 and 2.29 ± 0.05, respectively.

### 3.7. Stability Study

#### 3.7.1. Storage Stability of the Nanoemulsions

**Figure 6:**
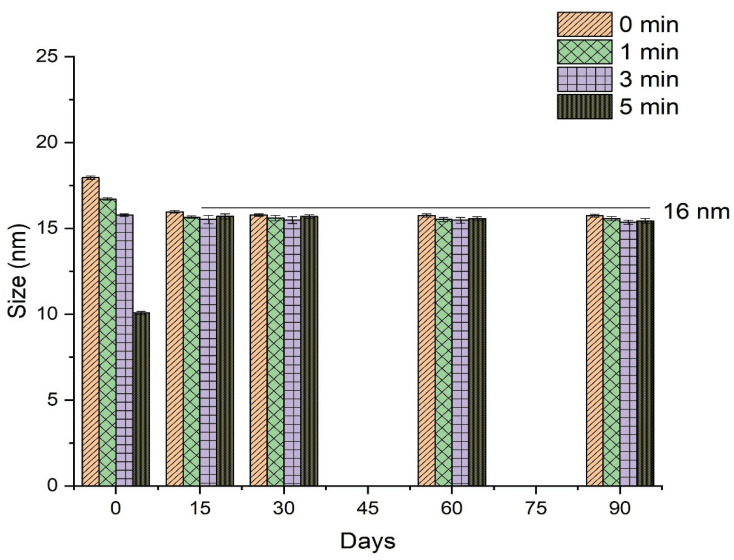
Effect of storage time on mean droplet size of retinyl palmitate-loaded nanoemulsions prepared under different vortexing conditions.

**Figure 7:**
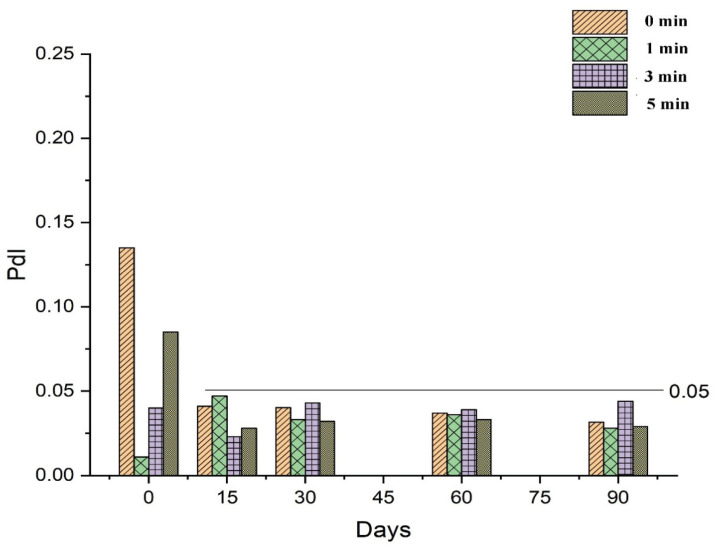
Effect of storage time on the polydispersity index (PdI) of retinyl palmitate-loaded nanoemulsions prepared under different vortexing conditions.

The stability of RT-loaded NEs prepared under different conditions (0, 1, 3, and 5 min of vortexing) was investigated during storage for 90 days by determining the droplet size and PdI throughout this time. As discussed in Section 3.2.1, the droplet size of freshly prepared NEs was influenced by variations in the vortex time, demonstrating an inversely proportional relationship as shown in Figure 6. Vortexing time also affected the PdI of the NEs, as a vortex time of 1 min decreased the value of PdI compared to no vortexing at all, while vortex times longer than 1 min progressively increased the value of PdI in freshly prepared samples, as shown in Figure 7. However, following 15 days of storage or more, values for mean droplet size and PdI remained relatively constant regardless of vortex time. These results indicate that NEs prepared by the low-energy method under different vortexing conditions reach an equilibrium stage after a specified period of time, after which the mean droplet size and PdI are no longer influenced by storage time.

#### 3.7.2. Physical Stability of RT-NEG

The physical stability of the RT-NEG was assessed following storage at ambient conditions (25 ± 2 °C and 75 ± 5% RH) for 90 days. The percentage of RT content, pH of the developed NEG, physical appearance, and viscosity of the formulation were evaluated at different time intervals (0, 30, 60, and 90 days) throughout the storage period. No significant changes (*p* > 0.05) were observed in any of these measurements throughout the storage period.

#### 3.7.3. UV Stability of RT and RT-NEG

**Figure 8:**
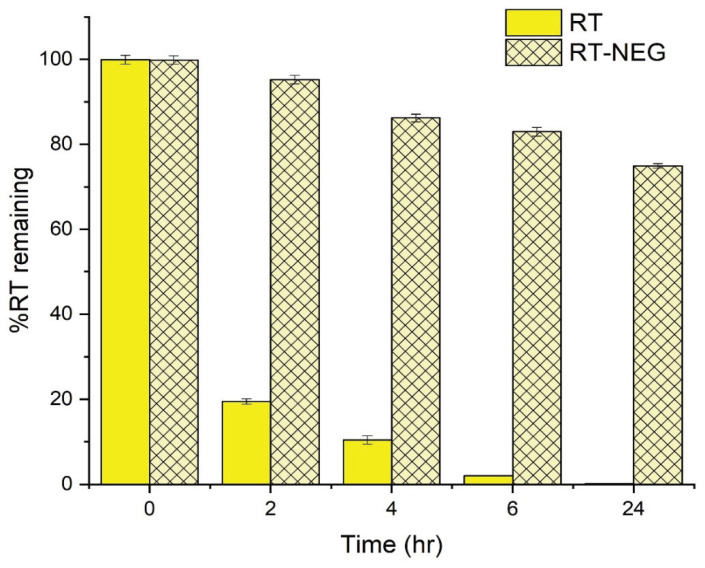
UV stability of pure retinyl palmitate (RT) and the RT nano-emulgel system.

The UV stability of the RT-NEG system and pure RT were investigated by measuring the percentage of RT remaining after exposure to UVA irradiation for varying time periods up to 24 h (Figure 8).

A significant decrease (*p* < 0.05) in the amount of pure RT was observed following 2 h of UV exposure, after which only 19.46% of the pure RT sample remained. In contrast, 95.24% of the RT in the NEG sample remained after 2 h of UV exposure. The pure RT sample was almost completely decomposed (2.02% remained) after 6 h of UV exposure, compared to 82.96% remaining in the NEG sample. The low stability of the pure RT sample is due to direct exposure of the polyunsaturated bonds to UVA radiation, resulting in decomposition [37]. RT encapsulated within an NEG system is shielded from direct interaction with UVA radiation, significantly improving the UV stability profile of this RT formulation compared to the pure sample.

## 4. Conclusions

The retinyl palmitate containing nano-emulgel system was successfully developed for their topical delivery exploiting low-energy emulsification technique. This investigation demonstrated that nano-encapsulation of nutraceutical/cosmeceutical/pharmaceutical showing poor biopharmaceutical performance and chemical/photo-instability resulting in improvement in UV and storage stability along with enhanced skin permeability after topical application. This improvement in results can be rationalized by superior solubilization ability of the nanoemulsion system, in addition to nano dimension of the encapsulating delivery vehicle favor the more permeation of retinyl palmitate into the skin through multiple mechanism/route of the epidermis. The investigation involved in this study demonstrated that control of HLB of the oil phase and vortexing duration in preparation of nanoemulsion of droplet dimension < 50 nm exploiting low-energy emulsification techniques are essential aspects for the topical delivery of hydrophobic nutraceutical/cosmeceutical/pharmaceutical into the skin.

## Figures and Tables

**Table 1:** Solubility of retinyl palmitate in different oils, surfactants, and co-surfactants.

Components	Solubility (mg/g)
Captex^®^ 355	> 1000
Capryol^®^ 90	305.12 ± 4.61
Transcutol HP	501.45 ± 4.57
Kolliphor^®^ EL	432.01 ± 3.80
Tween 20	128.76 ± 3.64

**Table 2:** Preformulation phase behavior study for the development of a retinyl palmitate-loaded nanoemulsion.

Oil Phase	Drug Loading	Smix Phase	Smix Ratio	Inference
Captex 355	Placebo	Tween 20 and Transcutol HP	1:1	Translucent emulsion
Capryol 90	Placebo	Tween 20 and Transcutol HP	1:1	Translucent emulsion
Captex 355	Placebo	Tween 20 and Transcutol HP	2:1	Translucent emulsion
Capryol 90	Placebo	Tween 20 and Transcutol HP	2:1	Transparent nanoemulsion >100 nm
Capryol 90 and Captex 355 (2:1)	Placebo	Tween 20 and Transcutol HP	2:1	Transparent nanoemulsion <100 nm
Capryol 90 and Captex 355 (2:1)	<15 mg/mL	Tween 20 and Transcutol HP	2:1	Transparent nanoemulsion <100 nm
Capryol 90 and Captex 355 (2:1)	>15 mg/mL	Tween 20 and Transcutol HP	2:1	Translucent emulsion
Captex 355	Placebo	Kolliphor EL and Transcutol HP	1:1	Translucent emulsion
Capryol 90	Placebo	Kolliphor EL and Transcutol HP	1:1	Transparent nanoemulsion >100 nm
Captex 355	Placebo	Kolliphor EL and Transcutol HP	2:1	Transparent nanoemulsion >300 nm
Capryol 90	Placebo	Kolliphor EL and Transcutol HP	2:1	Transparent nanoemulsion >300 nm
Capryol 90 and Captex 355 (2:1)	Placebo	Kolliphor EL and Transcutol HP	2:1	Transparent nanoemulsion <20 nm
Capryol 90 and Captex 355 (2:1)	50 mg/mL	Kolliphor EL and Transcutol HP	2:1	Transparent nanoemulsion >50 nm
Capryol 90 and Captex 355 (2:1)	40 mg/mL	Kolliphor EL and Transcutol HP	2:1	Transparent nanoemulsion >50 nm
Capryol 90 and Captex 355 (2:1)	30 mg/mL	Kolliphor EL and Transcutol HP	2:1	Transparent nanoemulsion <20 nm

**Table 3:** Formulation composition and characterization of retinyl palmitate-loaded nanoemulsions.

Formulation	Nanoemulsion Composition (%*w*/*w*)				%T	η (cp)	% Drug Content	Mean Droplet Size (nm)	PdI	ζ (mv)
	Oil	Kolliphor EL	Transcutol HP	Water						
NE1	20.0	30.0	15.0	35.0	97.84 ± 0.52	82.6 ± 1.61	99.06 ± 0.63	71.95 ± 1.46	0.606 ± 0.005	−19.03 ± 0.30
NE2	20.0	33.34	16.66	30.0	96.08 ± 0.31	89.22 ± 1.95	98.71 ± 0.72	45.24 ± 1.79	0.188 ± 0.015	−19.46 ± 0.40
NE3	15.0	33.34	16.66	35.0	98.51 ± 0.18	84.93 ± 1.52	98.87 ± 0.70	19.04 ± 0.17	0.120 ± 0.005	−20.06 ± 0.45
NE4	10.0	30.0	15.0	45.0	98.88 ± 0.03	77.48 ± 1.73	98.87 ± 0.55	16.71 ± 0.07	0.015 ± 0.001	−20.36 ± 0.49

**Table 4:** Characterization of the gel formulations from the skin permeation and deposition study.

Parameters	RT-NEG	RT-Gel	Placebo Gel	RT-Cream
Spreadability factor (cm^2^/g)	1.34 ± 0.03	1.27 ± 0.029	1.23 ± 0.034	-
Drug content uniformity (mg %)	98.72 ± 1.15	-	-	-
pH	5.53 ± 0.06	5.58 ± 0.02	5.55 ± 0.02	-
Drug deposited in skin (µg/cm^2^)	835.56 ± 19.69	204.29 ± 10.01	-	173.35 ± 6.29
Cumulative amount of drug permeated (µg)	417.30 ± 13.55	219.33 ± 14.52	-	184.11 ± 10.99
J_ss_ * (µg/cm^2^ h)	16.85 ± 0.4	8.54 ± 0.42	-	7.33 ± 0.47
Permeability coefficient (K_p_ × 10^−3^) **	3.37 ± 0.08	1.67 ± 0.03	-	1.46 ± 0.1
ER ***	1.97 ± 0.07	-	-	-

* Jss = Transdermal flux, calculated from the cumulative amount of drug permeated versus time. ** Permeability coefficient was calculated as Kp = Jss /C_0,_ where, C_0_ = the initial drug concentration in the donor compartment. *** ER = Enhancement ratio, ratio of transdermal flux from RT-NEG to RT-gel.

## Supplementary Materials

The following are available online at https://www.mdpi.com/2079-4991/10/5/848/s1, Figure S1: Droplet size (71.97 nm with PdI 0.619) and zeta potential (−19.1 mV) of NE1; Figure S2: Droplet size (45.46 nm with PdI 0.204) and zeta potential (−19.4 mV) of NE2; Figure S3: Droplet size (19.06 nm with PdI 0.125) and zeta potential (−20.1 mV) of NE3; Figure S4: Droplet size (16.63 nm with PdI 0.016) and zeta potential (−20.6 mV) of NE4.
