# Supplementary material for: Nanoemulgel for Improved Topical Delivery of Retinyl Palmitate: Formulation Design and Stability Evaluation
Source: Nanomaterials (Basel). 2020 Apr 28;10(5):848. doi: 10.3390/nano10050848 (PMC7711631; doi:10.3390/nano10050848)
Supplement: Supplementary file 1 [file nanomaterials-10-00848-s001.pdf]

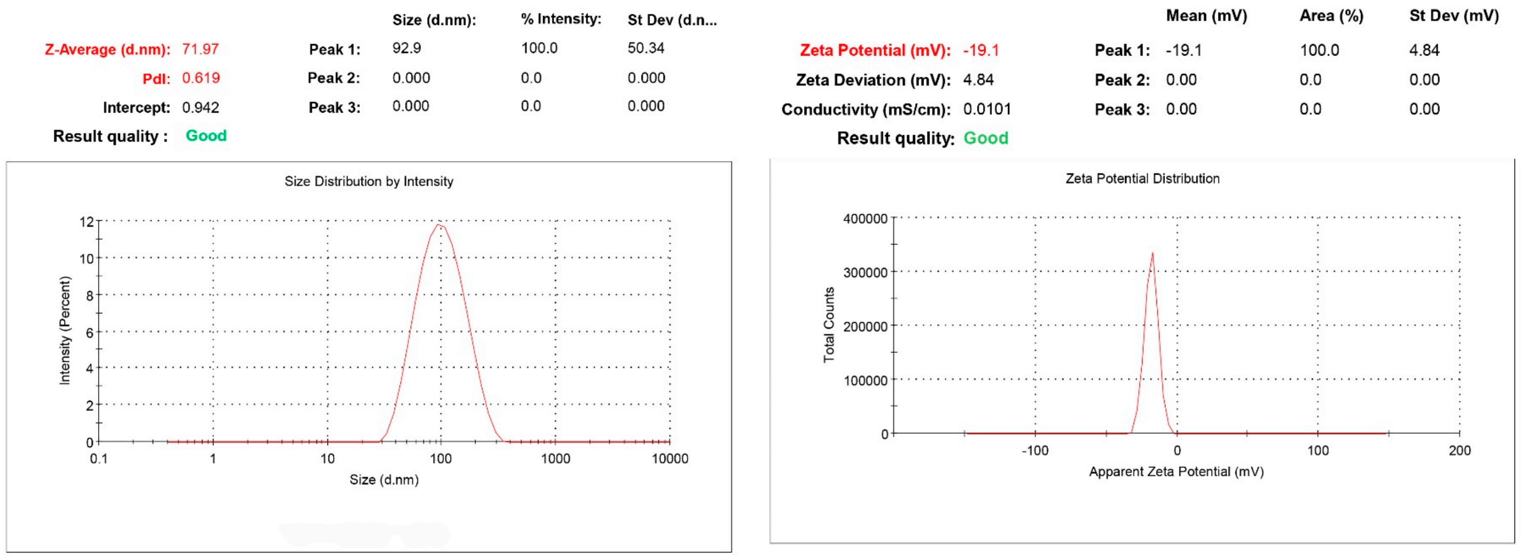

Figure S1: Droplet size (71.97 nm with PdI 0.619) and zeta potential (-19.1 mV) of NE1

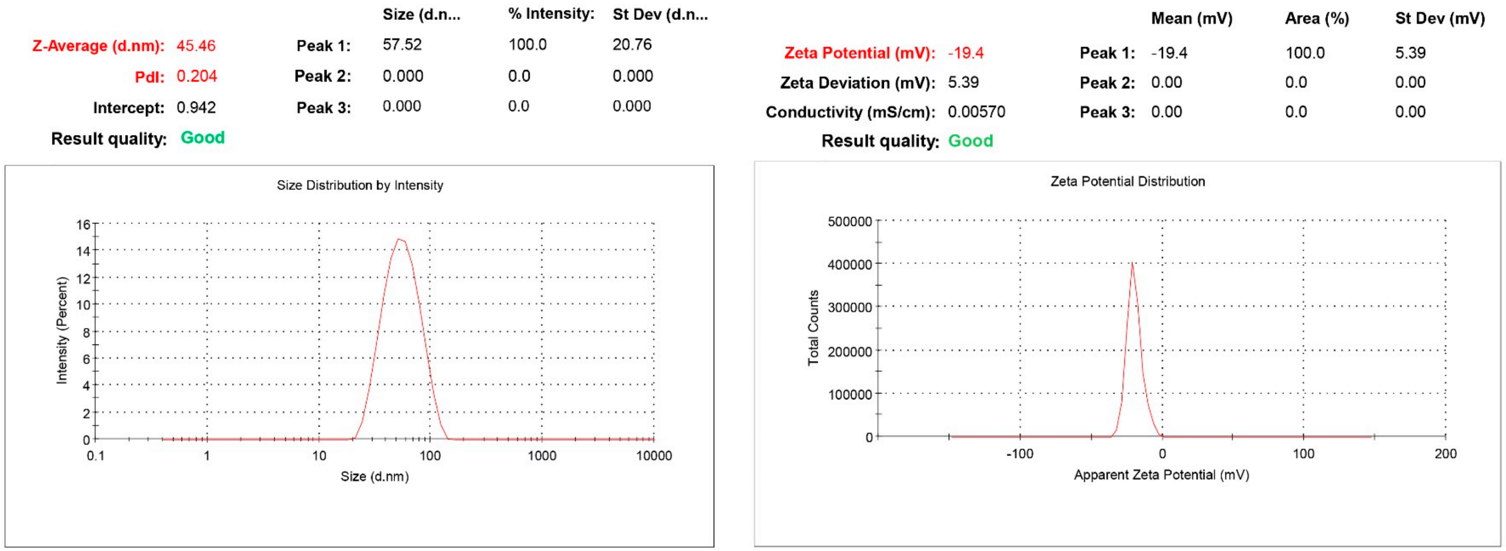

Figure S2: Droplet size (45.46 nm with PdI 0.204) and zeta potential (-19.4 mV) of NE2

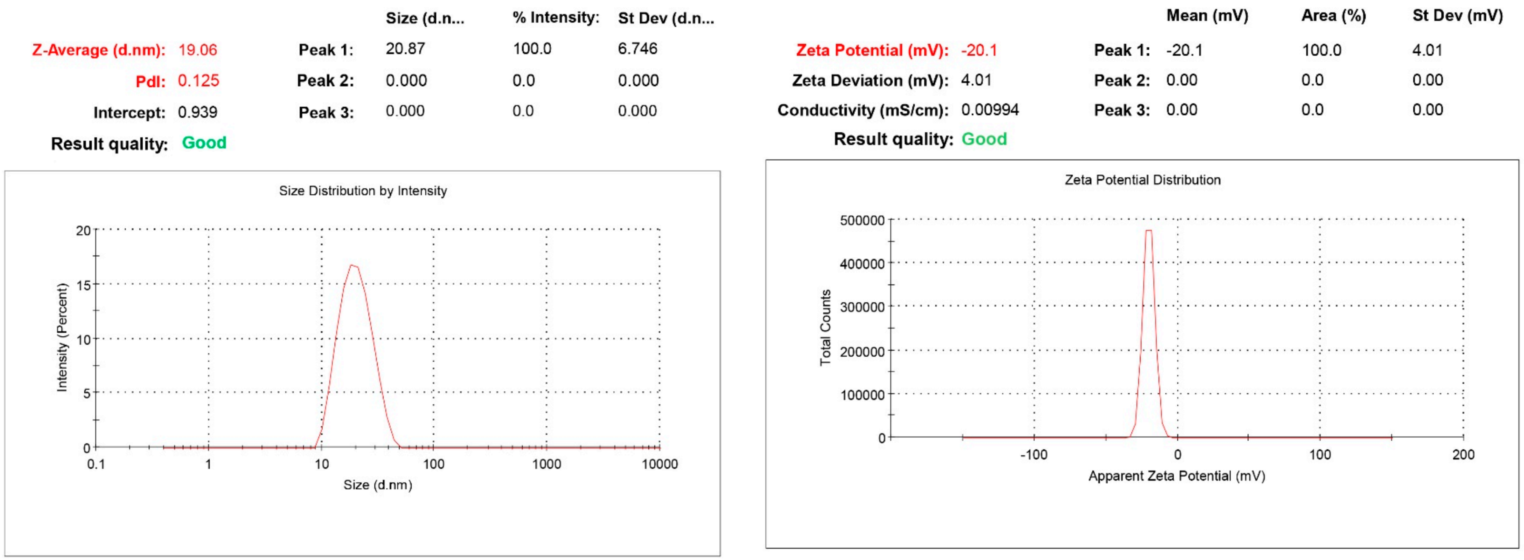

Figure S3: Droplet size (19.06 nm with PdI 0.125) and zeta potential (-20.1 mV) of NE3

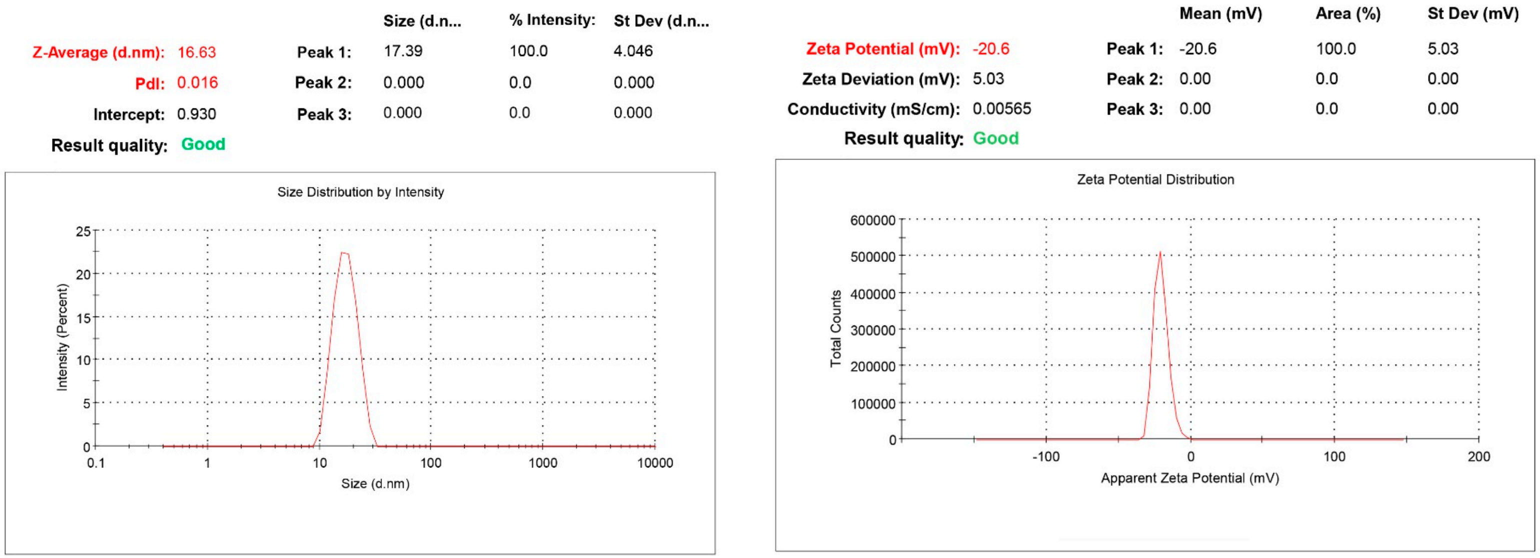

Figure S4: Droplet size (16.63 nm with PdI 0.016) and zeta potential (-20.6 mV) of NE4
